# Using social risks to predict unplanned hospital readmission and emergency care among hospitalized Veterans

**DOI:** 10.1111/1475-6773.14353

**Published:** 2024-07-07

**Authors:** Portia Y. Cornell, Cassandra L. Hua, Zachary M. Buchalksi, Gina R. Chmelka, Alicia J. Cohen, Marguerite M. Daus, Christopher W. Halladay, Alita Harmon, Jennifer W. Silva, James L. Rudolph

**Affiliations:** ^1^ Center of Innovation for Long Term Services and Supports Providence VA Medical Center Providence Rhode Island USA; ^2^ Centre for the Digital Transformation of Health University of Melbourne Melbourne Victoria Australia; ^3^ Department of Public Health, Zuckerberg College of Health Sciences University of Massachusetts Lowell Massachusetts USA; ^4^ National Social Work Program, Care Management and Social Work, Patient Care Services Department of Veterans Affairs Washington DC USA; ^5^ Tomah VA Medical Center Tomah Wisconsin USA; ^6^ Department of Health Services, Policy and Practice Brown University Providence Rhode Island USA; ^7^ Department of Family Medicine Alpert Medical School of Brown University Providence Rhode Island USA; ^8^ Rocky Mountain Regional Medical Center Aurora Colorado USA; ^9^ Gulf Coast Veterans Health Care System Biloxi Mississippi USA

**Keywords:** area deprivation index, emergency department use, food insecurity, hospital readmission, housing insecurity, social determinants of health, social isolation, social risk factors, Veterans

## Abstract

**Objectives:**

(1) To estimate the association of social risk factors with unplanned readmission and emergency care after a hospital stay. (2) To create a social risk scoring index.

**Data Sources and Setting:**

We analyzed administrative data from the Department of Veterans Affairs (VA) Corporate Data Warehouse. Settings were VA medical centers that participated in a national social work staffing program.

**Study Design:**

We grouped socially relevant diagnoses, screenings, assessments, and procedure codes into nine social risk domains. We used logistic regression to examine the extent to which domains predicted unplanned hospital readmission and emergency department (ED) use in 30 days after hospital discharge. Covariates were age, sex, and medical readmission risk score. We used model estimates to create a percentile score signaling Veterans' health‐related social risk.

**Data Extraction:**

We included 156,690 Veterans' admissions to a VA hospital with discharged to home from 1 October, 2016 to 30 September, 2022.

**Principal Findings:**

The 30‐day rate of unplanned readmission was 0.074 and of ED use was 0.240. After adjustment, the social risks with greatest probability of readmission were food insecurity (adjusted probability = 0.091 [95% confidence interval: 0.082, 0.101]), legal need (0.090 [0.079, 0.102]), and neighborhood deprivation (0.081 [0.081, 0.108]); versus no social risk (0.052). The greatest adjusted probabilities of ED use were among those who had experienced food insecurity (adjusted probability 0.28 [0.26, 0.30]), legal problems (0.28 [0.26, 0.30]), and violence (0.27 [0.25, 0.29]), versus no social risk (0.21). Veterans with social risk scores in the 95th percentile had greater rates of unplanned care than those with 95th percentile Care Assessment Needs score, a clinical prediction tool used in the VA.

**Conclusions:**

Veterans with social risks may need specialized interventions and targeted resources after a hospital stay. We propose a scoring method to rate social risk for use in clinical practice and future research.

**What is known on this topic**

- Many social risk factors are associated with higher rates of hospital readmission and emergency department use.
- The Department of Veterans Affairs currently does not have an index in widespread use to identify Veterans who experience heightened social risk.

**What this study adds**

- We organized comprehensive, structured data from the Veterans Affairs electronic health record into social risk domains and an overall social risk score
- We found that those social risks—alone and in combination—were strong predictors of unplanned care.
- Our algorithm can help identify Veterans at heightened risk and inform transitional care that centers on Veterans' needs.

## 1 INTRODUCTION

### 1.1 Background

Over 600,000 Veterans are discharged each year from a Department of Veterans Affairs (VA) medical center, and 19% are readmitted within 30 days at an average cost of $9000 per readmission.^1^ A bewildering array of medication changes, new self‐care responsibilities, and complex discharge instructions can present challenges to transferring care from the inpatient team to primary care and outpatient specialty care.^2^, ^3^ Breakdowns in any of those processes can contribute to an unplanned readmission or a visit to the emergency department (ED).^4^

Social risk factors such as financial hardship, social isolation, food insecurity, or unstable housing exacerbate the complexity of post‐acute care transitions. Patients who have experienced such conditions have a higher likelihood of unplanned readmission to the hospital^5^, ^6^, ^7^, ^8^, ^9^ and of ED visits.^4^, ^10^ In the VA, Veterans who live in high‐deprivation areas and those who report more social risk factors face a higher risk of hospital admission.^11^, ^12^, ^13^ Patients themselves are aware of the problem: one recent study found that almost half of people with a recent hospital admission said they needed general, nonmedical help to stay well and out of the hospital.^14^ Information on social risks can improve risk adjustment models, for example, in the Medicare Hospital Readmission Reduction Program.^15^, ^16^, ^17^ Other studies used data collected through interviews or surveys,^13^, ^18^ which provide useful information but are not feasible to implement at scale in the health system, and are not easily integrated with clinical data. In this analysis, we organized a comprehensive set of social risk variables collected during routine clinical care into conceptual domains and a clinically actionable risk score.

### 1.2 Conceptual framework and rationale

We conceptualized our analyses with the World Health Organization's (WHO) Commission on Social Determinants of Health framework.^19^ The WHO framework emphasizes the role of the health system itself as a social determinant of health by facilitating equitable access to care and promoting action by non‐health organizations and government to improve health status. Thus, the framework is well suited to informing a tool designed to prompt social care interventions in the VA health system. According to the framework, populations are stratified by social determinants of health such as income, education, occupation, race, and ethnicity. The social determinants affect outcomes by operating through what the WHO calls intermediate determinants, including material conditions (e.g., housing quality and neighborhood) and psychosocial determinants (e.g., social support, interpersonal violence). In keeping with current research terminology, we call these intermediate determinants “social risks.”^20^ Social risks are associated with the incidence and complications of health conditions such as cardiovascular disease, musculoskeletal conditions, and diabetes.^21^, ^22^, ^23^, ^24^, ^25^

### 1.3 Objective

Public health leaders have advocated for the creation of risk scores to capture patient‐level social risk for health outcomes and inform clinicians about which patients need social interventions to improve their health.^26^ There are several sources of data that can be used for this purpose, including clinician referrals and universal screenings in the VA for housing instability, food insecurity, and intimate partner violence. However, the VA does not have a nationally implemented tool to identify Veterans with social risk factors who may need assistance.

In this study, we identified sources of information on social risk factors in the VA health record and organized them into conceptual domains. In a sample of recently hospitalized Veterans, we assessed the association of those risk domains with hospital readmission and ED visits. From those models we developed a social risk score (SOS) designed to help clinicians prioritize assessment and social care toward Veterans who may need social support in care transitions.

## 2 METHODS

### 2.1 Study setting, data, and participants

This study was reviewed and approved by the Providence VA Medical Center Institutional Review Board and was conducted as part of evaluation and quality improvement activities of the VA National Social Work Patient Aligned Care Team (PACT) Staffing Program.^27^ We obtained data from the VA Corporate Data Warehouse, an administrative database of Veterans' health records.^28^ Data analysis was completed between September 5, 2023 and February 28, 2024. Eligible sites were 167 primary care clinics (including VA medical centers and community‐based outpatient clinics) that participated in the VA National Social Work PACT Staffing Program, about 15% of the VA's 1141 VA primary care sites.^27^ Sites were eligible for the program if at least 50% of Veterans who received primary care or social work services resided in a rural area. We identified all Veterans who had at least one primary care visit at sites that participated in the program between March 1, 2022 and February 28, 2023. Among these, we included Veterans who also had at least one inpatient medical or surgical hospital stay during that time in our analytic cohort. Veterans with more than one inpatient admission in the study period were observed multiple times in our data. Readmissions within 30 days were also analyzed as index stays. We used data from the Neighborhood Atlas on area deprivation.^29^

### 2.2 Outcomes

Our study included two primary outcome measures within 30 days of the index discharge date: unplanned readmission and any ED use. Care episodes at VA hospitals and VA‐paid care at community hospitals were included in the outcomes. We used the Agency for Healthcare Research and Quality definition to determine whether a readmission was planned or unplanned.^30^ We excluded planned readmissions defined as either a nonacute readmission in which a planned procedure occurred, or a readmission for maintenance chemotherapy and rehabilitation. If admissions occurred within 24 h, we combined them into a single episode to exclude potentially erroneous readmissions that were continuations of the index stay but not documented properly in the health record.

### 2.3 Identification of social risk domains

We coded the social risk data using techniques derived from deductive content analysis.^31^ We identified structured categories of health‐related social risk factors from previous work on Veterans,^4^, ^13^, ^32^, ^33^ the Social Interventions Research and Evaluation Network screening tool comparison table,^34^ the Centers for Medicare and Medicaid services Accountable Health Communities screening tool,^35^ and ICD‐10 guidance.^36^ We noted VA operational social risk domains used in the social work comprehensive assessment note template (i.e., access to care, economic, housing, social support, psychological, and functional status), and the VA Assessing Circumstances and Offering Resources for Needs (ACORN) social needs screening tool^37^ (i.e., housing instability, food insecurity, utility needs, lack of transportation, social isolation/loneliness, interpersonal violence, legal assistance, educational needs, and employment concerns).

We then narrowed this list based on the WHO's framework to focus on domains that (1) are intermediate social risk factors, and (2) represent needs addressable through healthcare system or by referral to community resources. For usability, we combined some categories. For example, employment, monetary, and education concerns comprise the “financial” category; elder abuse, intimate partner violence, physical abuse, and trauma‐related diagnoses were combined as “violence;” and caregiver needs were included in “social support.” Taxonomies of social risk usually consider mental and behavioral health as clinical rather than social conditions, therefore we did not include mental or behavioral health diagnoses in the main model. However, Veterans with mental health conditions disproportionately experience social risks and social and psychological interventions are also critically important to their care.^38^ Therefore, we reported a version of the models with a “mental/behavioral” domain in an appendix. Thus informed by the WHO's schema, the final nine categories included material circumstances (food, housing, financial, transportation, and neighborhood deprivation), psychosocial factors (social support, legal, and violence), and nonspecific factors (diagnoses or assessments suggesting undefined or multiple complex social problems). The nine domains are listed in Table 1.

**TABLE 1:** Social risk domains, description, and examples of data elements.

Social risk domain	No. observations (%)	Description	Example health factors	Example ICD‐10 codes	Example stop codes
Nonspecific psychosocial	24,219 (46.7)	Unspecified or need for social work case management	SOC WORK CASE MGMT LEVEL 4—INTENSIVE	Z65.8 Other specified problems related to psychosocial circumstances	n/a
Financial	11,275 (21.8)	Hardship related to income, employment, or unemployment	SOC WORK PRESENTING ISSUE‐FINANCIAL	Z56.0 Problems related to employment/unemployment	208/222/568/574 Compensated work therapy
Social support	9004 (17.4)	Problems related to social environment, primary support group, social isolation, loneliness, caregiver stress	SOC SUPPORTS‐NONE	Z60 Problems related to social environment	n/a
Housing	7646 (14.8)	Homelessness, housing insecurity, difficulty getting access to or paying for utilities	SOC WORK PRESENTING ISSUE‐HOUSING	Z59.0 Homelessness	508/528/529 Health Care for Homeless Veterans
Food insecurity	3067 (5.9)	Limited/uncertain access to adequate nutritional food	VA‐MONEY TO BUY FOOD‐YES CURRENT CONCERN	Z59.41 Food insecurity	n/a
Access to care	2766 (5.3)	Problems related to transportation or difficulty accessing care	SOC WORK PRESENTING ISSUE‐ACCESS TO CARE	Z59.82 Transportation insecurity	n/a
Legal	2180 (4.2)	Problems related to incarceration or legal	SOC WORK PRESENTING ISSUE‐LEGAL	Z65.0 Conviction in civil and criminal proceedings without imprisonment	591 Incarcerated re‐entry
Violence	2033 (3.9)	Elder abuse, neglect, intimate partner violence (user or survivor), trauma, violent conflict	SOC WORK PRESENTING ISSUE‐ABUSE/NEGLECT	T7401XA, Adult neglect or abandonment, confirmed, initial encounter Z04.41 Encounter for examination and observation following alleged rape	524 Active duty sexual trauma
Neighborhood deprivation	1530 (3.0)	National Area Deprivation Index (ADI) ≥96	n/a	n/a	n/a

Abbreviation: ICD, International Classification of Disease.

### 2.4 Exposures

To populate these domains with observed data, we created a list of potential social risk identifiers from four sources: ICD‐10 codes, stop codes, health factors, and geocodes. First, we identified ICD‐10 codes potentially related to social risks, including all “Z” codes and others related to trauma, abuse, or social circumstances.^39^ Second, based on input from VA clinicians (i.e., social workers, registered nurses, primary care physicians, and hospitalists) we identified a list of VA “health factors” related to social determinants. Health factors are how structured clinical data are stored and processed in the VA electronic health record. They include most clinical reminders (such as housing and food insecurity and partner violence) and certain standardized assessments, completed by social workers assigned to a primary care PACT. For example, a social worker's comprehensive assessment generates structured data that the Veteran reports difficulty rated by the social worker from level 1 (“generally has their personal needs met”) to level 4 (“crisis.”) In our data, a social work assessment is designated a positive within that domain if the level is 2 (“minor concerns”) or higher. In the “Access to Care” domain, for example, according to guidance from the VA National Social Work Program, problems that would prompt concern typically would include inquiring about other sources of health insurance or care, needing assistance with eligibility or transportation, applying for Medicaid, coordinating an appointment, or arranging temporary lodging coinciding with a scheduled appointment or procedure. The VA instituted universal screenings in 2012 for housing insecurity and in 2017 for food insecurity for all non‐institutionalized Veterans receiving outpatient primary care.

Third, we included VA service “stop” codes associated with various types of clinical encounters, for example, services related to housing, employment, and military sexual trauma. Finally, we used geocodes for Veterans' home addresses to link them to the Area Deprivation Index (ADI).^29^ We operationalized “high deprivation” neighborhoods as those in the national 95th percentile. Examples of how these data elements were used to assign social risk domains are shown in Table 1. A full list of ICD‐10 codes, health factors, and stop codes and their assigned social risk domains is included in the online supplement materials.

Each data element was coded as follows: three members of the research team independently reviewed the complete list of variables and either indicated the domain they thought it best fit or recommended excluding that variable. If the variable could match to more than one domain, the researchers ranked their first and second choice domains. The categorized codes were compiled and any variables with discrepancies flagged for discussion. The group met to review codes and achieved consensus on the variables' assignment.

Each social risk domain was operationalized as a binary variable equal to 1 if the Veteran had any of the identified social risks in their health record in the 12 months up to and including the day of discharge from the hospital stay, and 0 if none of the data elements were observed or reported in that period.

### 2.5 Covariates

Demographic characteristics included age (5‐year bins) and sex assigned at birth. To adjust for medical factors associated with risk of readmission, we calculated each person's HOSPITAL score (Hemoglobin at discharge, discharge from an Oncology service, Sodium level at discharge, Procedure during the index admission, Index Type of admission [non‐elective], number of Admissions during the last 12 months, and Length of stay).^40^ Because the VA Corporate Data Warehouse does not record discharge from oncology service, we instead used cancer as the primary discharge diagnosis for the purposes of calculating HOSPITAL score. To identify non‐elective admissions, we applied the same algorithm we used to identify the unplanned readmission outcome.

In addition to the variables used in model adjustment, we also reported descriptive characteristics of Veterans' race (Asian, American Indian or Alaska Native, Black or African American, Native Hawaiian or Other Pacific Islander, White, more than one race, and unknown), VA priority enrollment group (Service‐connected disability, 1 and 2; wartime service, 3; other disability, 4; Low‐income, 5 and 7; other groups, 6 and 8), and rurality using the VA's designation of rurality, which uses the following Rural Urban Commuting Area (RUCA) classifications (urban, RUCA = 1, 1.1, 30% or more in metropolitan area; highly rural, RUCA = 10.0, 10% or less of the population commutes to urban areas; and rural, all tracts not defined as urban or highly rural). Our measure of race represents a social construct, used as a proxy to measure the influence of structural racism experienced by Veterans in our study.^41^ Race information is collected through clinical interactions, and though efforts are made to collect self‐reported race information in VA, race information is often recorded by a proxy respondent or a VA enrollment coordinator or clerk.^42^

### 2.6 Analyses

All analyses were conducted using R x64 4.1.2. We reported the demographics, clinical characteristics, and prevalence of social risks among the hospital‐discharge cohort with sample means overall and stratified by Veterans who were identified as having one or more social risk factors compared to those who had no identified social risks using means, standard deviations (SD), and standardized mean differences (SMDs). The standardized mean difference is the difference in means divided by the pooled, weighted standard deviation (Cohen's *d* and Cohen's ω, respectively, for continuous and categorical variables).^43^

We reported the rate of each outcome among Veterans with a given social risk domain and those with no social risks. To calculate risk‐adjusted probabilities, we estimated multivariable logistic regression adjusting for age (categorized in 5‐year bins), sex at birth, HOSPITAL score (fixed effects), and indicators for each social risk domain. We did not include race, rurality, or VA priority enrollment status in the adjustment because in our conceptual model these characteristics were mediators or drivers of the relationship of social risk to health. We identified statistically significant differences based on a Wald test of the logistic regression coefficients, but to facilitate intuitive interpretation^44^ we reported average marginal probabilities and 95% confidence intervals [CIs] (calculated with R “modmarg” package) for Veterans in each social risk group, compared to those with no social risk.

We created a SOS to represent a Veteran's social risk factors weighted by their association with unplanned care. We estimated logistic models for both outcomes from the social risk domains as predictors. Within the conceptual framework, health conditions mediate the relationship between social risks and healthcare use. Thus, we did not adjust for age, sex, or clinical factors in the weight calculation, as controlling for clinical risk would diminish the association between social risks and unplanned care. On a practical level, conditioning on clinical factors would then create an index that would need to be interpreted simultaneously with clinical risk prediction. We anticipated that a standalone score would increase the usability of the score and facilitate its integration into care planning. Domains with negative coefficients on first estimation were excluded from the index, and the final models were re‐estimated on the smaller group of domains. Although excluding domains with negative coefficients may lead to decrease in overall predictive performance of the SOS, negative coefficients may indicate that Veterans use less hospital care because of a lack of access, and we did not want to index to assign lower priority to individuals with social risks that may represent barriers to care. We calculated a weight for each domain by adding coefficients from the two models, a technique that has been used previously to create risk algorithms.^40^, ^45^ For reporting purposes, we rescaled the log‐odds coefficients to sum to 100. For each Veteran, their raw SOS was the sum of weights for social risks present at the time of discharge (i.e., recorded in the health record within the previous 12 months). We then converted the score to a percentile rank from 0 to 99, creating a scale similar to the Care Assessment Needs (CAN) used widely in VA to predict hospitalization and death.^46^ To examine the performance of the score, we plotted 30‐day readmission and ED use against the SOS and CAN percentiles using a local polynomial smooth kernel function. We used the most recent CAN score as of the Veteran's discharge date. Using a 95th percentile cutoff value, we calculated the positive predictive value (out of cases with score ≥95% with the outcome), sensitivity (percent with score ≥95 out of Veterans with the outcome), and specificity (of Veterans who did not experience the outcome, percent of with score <95). To assess the model for potential bias, we compared these model performance metrics by birth, sex, and race.

## 3 RESULTS

The final analytic sample included 51,832 Veterans. Table 1 shows the prevalence of each social risk. The domains with the highest prevalence in our cohort were nonspecific psychosocial (46.7%), followed by financial (21.8%), social support (17.4%), and housing (14.8%). The full list of data elements assigned to each category is available in the online supplement.

Table 2 shows descriptive statistics on Veterans' characteristics in this cohort. The mean age was 67.8 (SD 14.2) and 6.2% were female. Among these, 73.9% Veterans had one or more indicators of social risk within the previous 12 months. Compared to Veterans with no social risk, Veterans who had experienced a social risk were younger (66.6 vs. 71.1 years, SMD = 0.34), more likely to be Black or African American (20.1% one or more risk vs. 12.6% no risks), and more likely to have a VA enrollment priority with a service‐connected or other disability (55.4% vs. 45.1%), and more likely to live in an urban area (56.5% vs. 49.0%). Veterans with social risks were also clinically more complex, with higher mean HOSPITAL scores than Veterans with no social risks (4.67 vs. 3.85, SMD = 0.45).

**TABLE 2:** Characteristics of Veterans in sample at time of discharge from a Veterans Affairs medical center hospital.

	Overall	No social risks	1+ social risks	Standardized mean difference^c^
No. of observations	51,832	13,526	38,306	
Age (mean [SD])	67.76 (14.16)	71.12 (12.08)	66.57 (14.64)	0.339
Sex				
Male (%)	48,604 (93.8)	12,994 (96.1)	35,610 (93.0)	0.137
Female (%)	3228 (6.2)	532 (3.9)	2696 (7.0)	
Race (%)				0.213
American Indian or Alaska Native	545 (1.1)	101 (0.7)	444 (1.2)	
Asian	195 (0.4)	88 (0.6)	107 (0.3)	
Black or African American	9377 (18.1)	1705 (12.5)	7672 (20.1)	
More than one race	548 (1.1)	110 (0.8)	438 (1.1)	
Native Hawaiian or other Pacific Islander	300 (0.6)	74 (0.5)	226 (0.6)	
Unknown	2535 (4.9)	666 (4.9)	1869 (4.9)	
White	38,332 (74.0)	10,881 (79.9)	27,451 (71.8)	
VA priority enrollment (%)				0.283
Service‐Connected Disability (1, 2)	25,983 (50.1)	5813 (43.0)	20,170 (52.7)	
Wartime Service (3)	5159 (10.0)	1662 (12.3)	3497 (9.1)	
Other Disability (4)	1723 (3.3)	287 (2.1)	1436 (3.7)	
Low‐Income (5, 7)	13,901 (26.8)	3772 (27.9)	10,129 (26.4)	
Other (6, 8)	5031 (9.7)	1984 (14.7)	3047 (8.0)	
Missing	35 (0.1)	8 (0.1)	27 (0.1)	
Rurality^a^				0.17
Highly rural	3225 (6.2)	1084 (8.0)	2141 (5.6)	
Rural	20,328 (39.2)	5815 (43.0)	14,513 (37.9)	
Urban	28,279 (54.6)	6627 (49.0)	21,652 (56.5)	
HOSPITAL score (mean [SD])^b^	4.46 (1.92)	3.84 (1.75)	4.67 (1.93)	0.454
Unplanned readmission 30 days	3813 (7.4)	701 (5.2)	3112 (8.1)	0.132
ED use 30 days	12,388 (23.9)	2689 (19.9)	9699 (25.3)	0.156

Abbreviations: ED, emergency department; SD, standard deviation.

^a^ Rurality is defined by Rural Urban Commuting Codes (RUCA) groups: Urban, RUCA = 1, 1.1, 30% or more in metropolitan area; Highly Rural, RUCA = 10.0, 10% or less of the population commutes to urban areas; Rural, all tracts not defined as Urban or Highly Rural.

^b^ HOSPITAL score is a weighted score based on Hemoglobin at discharge, discharge from an Oncology service, Sodium level at discharge, Procedure during the index admission, Index Type of admission, number of Admissions during the last 12 months, and Length of stay.

^c^ The standardized mean difference is the difference in means divided by the pooled, weighted standard deviation (Cohen's *d* and Cohen's ω, respectively, for continuous and categorical variables).

**FIGURE 1:**
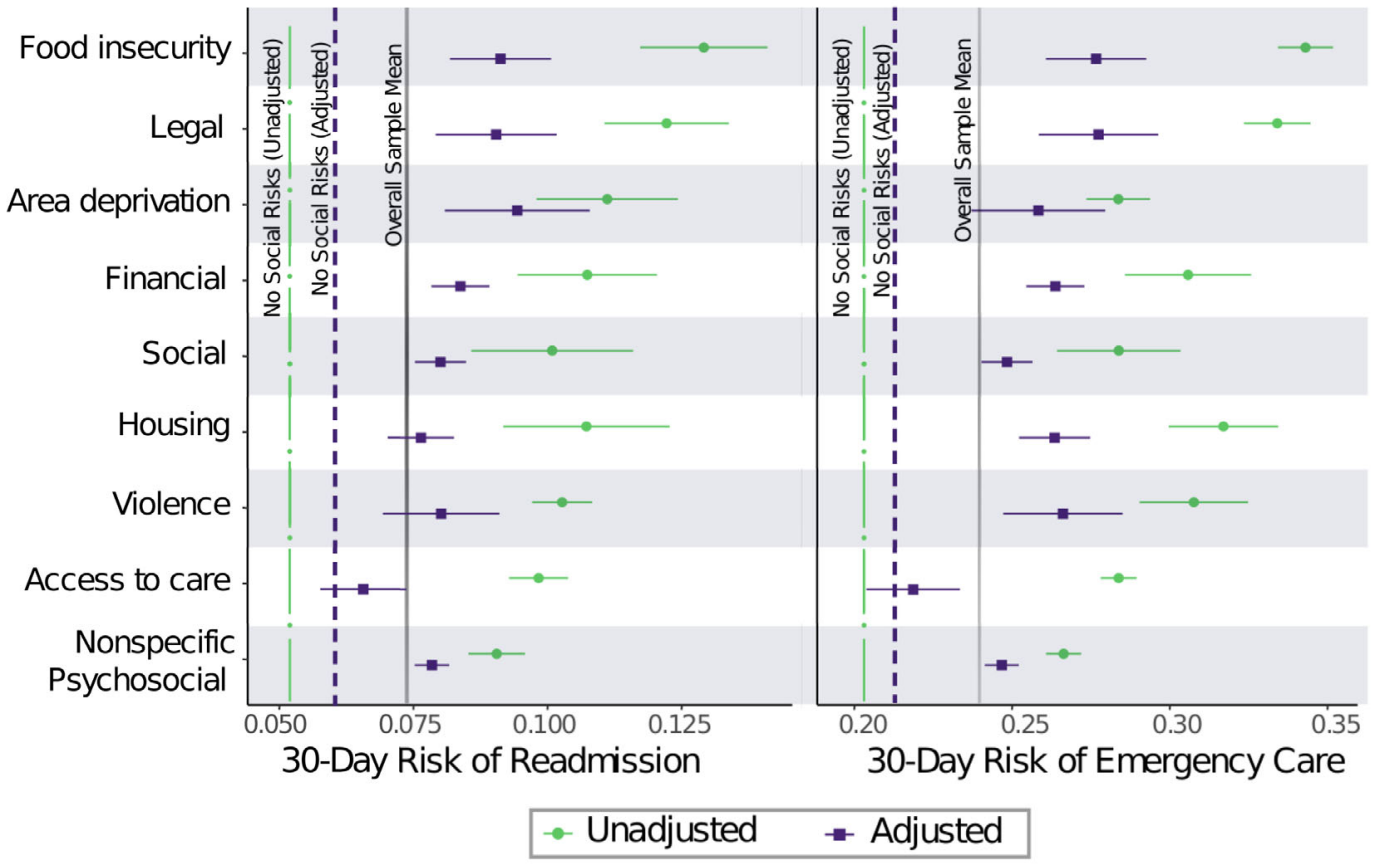
Veterans with social risks have higher probabilities of unplanned readmission and emergency department use than those with no social risk. Figures show point estimates and 95% confidence intervals of population‐average predicted margins from multivariable logistic regression models. Adjusted outcomes are modeled with social risk domains, age, sex, and HOSPITAL score. HOSPITAL score is a weighted score based on Hemoglobin at discharge, discharge from an Oncology service, Sodium level at discharge, Procedure during the index admission, Index Type of admission, number of Admissions during the last 12 months, and Length of stay.

Overall, the 30‐day unplanned readmission probability was 0.074 (Table 1). Veterans with one or more social risks had over 50% greater probability of 30‐day unplanned readmission than those without (0.081 vs. 0.052). Figure 1 shows the unadjusted rates and adjusted rates as predicted outcome risks among Veterans reporting each social risk within the previous 12 months. We report full model estimates in the Table S2. Unplanned care risks were greatest among Veterans with food insecurity (0.129), legal need (0.124), and neighborhood deprivation (0.111). After adjusting for age, sex, and HOSPITAL score, Veterans with no social risks had an adjusted probability of readmission of 0.052. Veterans with the greatest adjusted probabilities of readmission were those with food insecurity (adjusted probability = 0.091, 95% CI [0.082, 0.101]), legal need (adj. pr. = 0.090 [0.079, 0.102]), and neighborhood deprivation (adj. pr. = 0.081 [0.081, 0.108]). Financial need, social isolation, nonspecific psychosocial, and housing were also associated with higher rates of readmission than having no social risks, ranging from 0.066 to 0.080. These were 50% to 150% greater than the adjusted risk among Veterans with no social risks (adj. probability = 0.052). Adjusted associations of violence, housing, and access to care with outcomes, compared to not having these risk factors, were not statistically significant. See Table S1 for coefficient estimates and statistical tests.

The overall risk of any ED use in 30 days was 0.239. Veterans with any social risk were more likely than those with none to have at least one ED visit (0.253 vs. 0.199). The greatest unadjusted risk of ED use was among Veterans with food insecurity (probability = 0.34), legal need (pr. = 0.34), and housing (pr. = 0.32), followed by violence (pr. = 0.31) and financial problems (pr. = 0.31), with these groups having more than 50% greater probability of ED use relative to Veterans with no social risks (pr. = 0.199). Adjusting for sex, age, and HOSPITAL score, the greatest risks of ED use were among Veterans who had experienced food insecurity (adj. pr. = 0.28 [0.26, 0.30]), legal problems (adj. pr. = 0.28 [95% CI 0.35, 0.29]), violence (adj. pr. = 0.27 [0.25, 0.29]), area deprivation (adj. pr. = 0.26 [0.24, 0.28]), financial need (adj. pr. = 0.26 [0.25, 0.27]), and housing problems (adj. pr. = 0.26 [0.25, 0.27]). In comparison with Veterans with no social risks (adj. pr. = 0.21), Veterans with these social risks had 24% to 33% greater adjusted probability of ED use. Veterans with social support needs and nonspecific psychosocial problems were also significantly more likely to have any ED visits (adj. pr. = 0.25 [0.24, 0.26]). Veterans with Access to Care‐related issues had a lower adjusted probability of an ED visit compared to Veterans without access to care problems, and no detectable difference from Veterans with no social risk.

In an alternative analysis we included a mental/behavioral health domain (see Tables S4 and S5). Mental and behavioral health were substantially associated with both outcomes but were not the strongest social‐risk predictors.

**FIGURE 2:**
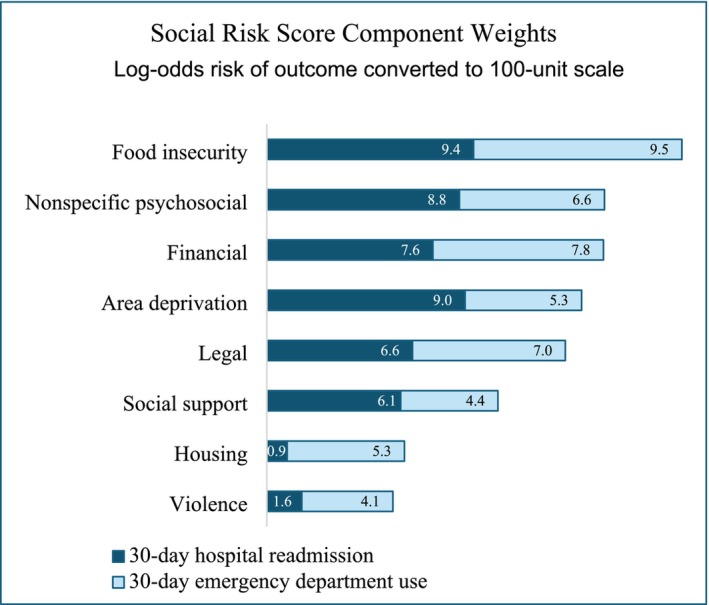
Final model weights on social risk domains used to calculate social risk score. Weights are derived from log‐odds coefficients estimated from logistic regression models of the probability of 30‐day unplanned readmission and emergency department care. For each domain, coefficients were summed for the two models. Then all weights were re‐scaled to sum to 100. Model coefficients and average marginal effects are reported in the online supplement.

**FIGURE 3:**
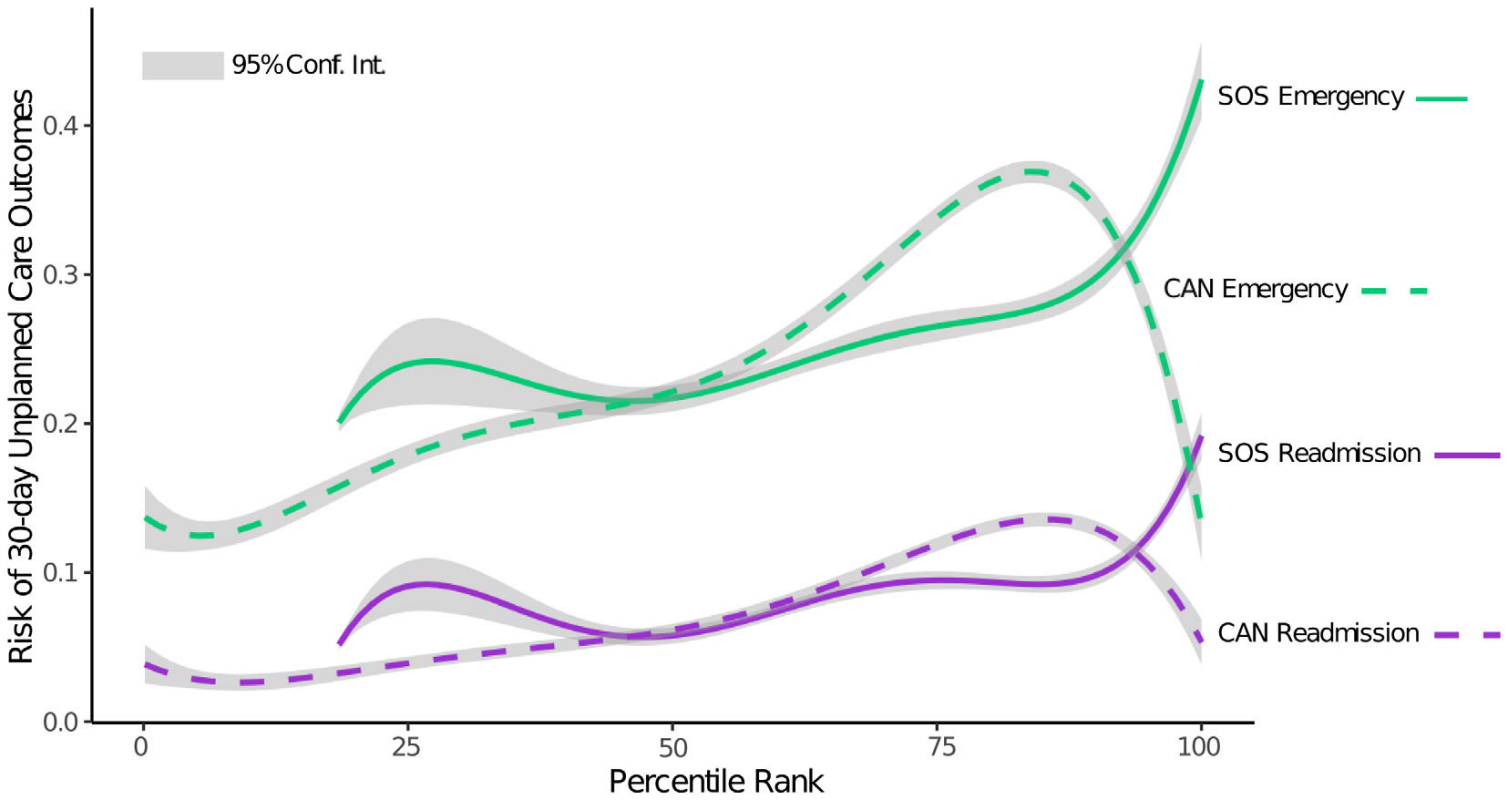
Risks of unplanned readmission and emergency care increase with higher Social Risk Score (SOS). SOS the percentile rank representing a Veterans' probability of hospital readmission and emergency department use based on social risk factors. Care Assessment Needs (CAN) is the percentile rank representing risk of all‐cause hospital admission or mortality in 1 year.

The final models used to compute weights for the SOS included all the social risk domains except access to care, for which the coefficients were negative for one or both outcomes. Figure 2 shows the SOS weights and the relative contribution of hospital readmission and ED outcomes. After re‐scaling, the weights ranged from 5.7 (violence) to 19.1 (food insecurity). Model coefficients and average marginal effects are reported in the online supplement. Figure 3 shows unplanned readmission and ED use plotted against the percentile rank of SOS and CAN score. From percentiles 25 to 99, risk of both readmission and ED use increase monotonically with higher SOS. By comparison, the mean outcomes increase more steeply for CAN percentiles 25 to 75, but above CAN of 80 the readmission and ED risk decrease steeply. The SOS at the 95th percentile had greater positive predictive value than CAN score ≥95: high‐SOS Veterans have higher risk of unplanned care (readmission 0.153, ED use 0.379) than high‐CANs Veterans (readmission 0.069, ED use 0.110). Using 95 as the cutoff score, the sensitivity (percent Veterans experiencing the outcome who were above the cutoff) of SOS is 10.6% versus 3.0% for CANs. For ED use, SOS had sensitivity of 37.9%, versus 11.0% for CANs. The greater sensitivity of the SOS compared to CANs was consistent across race groups and sexes (see Figure S2). Using a 5‐fold cross‐validation, we found that the SOS model performed consistently when predicting out of sample (Table S6). When we examined model performance by race and sex, the model had the greatest sensitivity for American Indian or Alaskan Native, Native Hawaiian or Other Pacific Islander, Black and multi‐race groups, and had lower sensitivity for females than males (see Figure S3). When comparing high‐SOS to low‐SOS Veterans in the sample, we found that a greater proportion of high‐SOS Veterans were female, American Indian or Black, in a low‐income VA priority group, or lived in an urban area (Table S8).

## 4 DISCUSSION

In the VA, Veterans' social risks are routinely identified through clinical screenings, diagnosis codes, social work assessments, and service codes. In our analyses of over 50,000 Veterans, 74% of recently hospitalized Veterans were identified to have one or more social risks. We organized social risk data into nine domains. The most prevalent domains were nonspecific psychosocial, financial need, and social support. Veterans at greatest risk for hospital readmission were those who had experienced food insecurity, legal problems, financial need, or area deprivation in the prior year. The greatest risk of ED use was among Veterans who had experienced food insecurity, legal problems, housing insecurity, or violence.

We proposed a SOS representing Veterans' risk of unplanned care related to social factors. We observed substantial increase in the risk of unplanned care above the 90th percentile. The nonlinearity of the SOS with outcomes has been found in other studies of social risk; for example, the association of Area Deprivation Index and readmission in one study increased sharply at the 85th–90th percentiles.^6^ High‐SOS Veterans (95th percentile) had greater levels of unplanned care than Veterans with a high CAN score, a tool used across VA representing risk of mortality and hospitalization. CAN is used in the VA model to target care‐coordination resources to Veterans with complex medical needs.^47^ The comparison of SOS percentiles to CAN percentiles suggests that Veterans who need social intervention might not be flagged with the use of medical risk alone.

### 4.1 Interpretation and research context

Our study builds on a rapidly growing body of evidence of the contribution of social risks to unplanned care and is novel in two important ways. First, the approach synthesizes previous studies and represents a comprehensive compendium thus far of structured social risk data in the VA. Second, we propose a numerical score summarizing a Veteran's social risk. The nine domains were motivated and constructed from a conceptual framework, and their relative weights were determined empirically to inform intervention and clinical practice.

The first group of domains represents material needs: food, housing, neighborhood, and access to care. Food insecurity and housing instability are associated with increased ED use and hospitalization.^48^, ^49^, ^50^ Food insecurity among Veterans has also been shown to be a risk factor for suicidal ideation^51^ and is associated with diabetes, depression, a number of trauma‐related co‐morbidities, as well as other social risks including housing instability.^52^ Homeless Veterans have been recognized as persistent “super‐users” of acute care services.^53^ The VA has a substantial commitment to providing care for homeless Veterans that addresses their social needs.^54^, ^55^ The extensive support for homeless Veterans may explain why we find housing insecurity to have an association with unplanned care, but not as strong a predictor as some other social risk domains. Additionally, prior researchers have hypothesized that homeless Veterans are more likely to be discharged to post‐acute care facilities, decreasing their probability of returning to the ED or hospital within 30 days^56^—a hypothesis that merits further investigation.

Consistent with many studies, the Area Deprivation Index (ADI) was associated with hospitalization and readmission in our analyis.^6^, ^7^, ^23^, ^57^, ^58^ However, to our knowledge, this research was the first to examine ADI alongside person‐level social risk information derived from screenings and diagnoses. In contrast with our finding, one study in a non‐Veteran population did not detect that neighborhood deprivation improved prediction of ED use.^59^ Because neighborhood deprivation can be calculated for any Veteran with a valid address in their VA record, it is potentially valuable in identifying social precarity among Veterans who have not received screening due to low engagement with the health system.

In adjusted models, we did not detect a positive association of Access to Care problems with our unplanned care outcomes, in contrast with a prior study that found lack of transportation significantly increased probability of ED use.^10^ This finding may be in part because difficulty with access hospital decreases healthcare use.

The second group of domains include psychosocial components: social support, social isolation, interpersonal violence, and legal problems. In the literature, low social support and lack of in‐home support are associated with readmission.^56^, ^60^ Our models also indicated that psychosocial factors were predictive of unplanned care needs.

Prior work has identified the “nonspecific psychosocial” diagnosis code as a strong correlate of mortality and suicidal ideation.^32^, ^33^ The strength of this domain as a predictor of health and health service use in VA may be due to the way clinicians use this diagnosis code to indicate myriad complex social problems. A qualitative study examining the perceptions and intentions behind use of this diagnostic would be an important future contribution to research.

### 4.2 Limitations

Our study should be interpreted considering its limitations. As an integrated health system, the VA is superlative in both its screening and resources for social care. While estimates as to the prevalence of social risk factors and signal from social‐risk data may not be generalizable outside the VA, it can serve as a model for embedding social care in the health system. Perhaps because they are not tied to financial incentives, use of social diagnosis codes outside the VA is comparatively low, with estimates ranging from less than 1% among Medicare beneficiaries^61^ to one in six in a dataset of surgical patients^62^—though the trend is increasing. Furthermore, within VA, because the sample is drawn from participants in the VA National Social Work PACT Staffing Program, our study may include a slightly higher proportion of rural Veterans, compared to a nationally representative sample of hospitalized patients. Additional work is needed to validate the model in other samples of Veterans.

Any social risk tool is only as good as the screening and assessment processes that produce the data—and those data are far from complete. In this study, we included any social risks noted in the patient's record, by any clinician, up to 12 months prior to their hospital discharge because social stressors are often ongoing; but, a social risk identified months ago may have been resolved or the Veteran's needs met with respect to that issue. Clinicians may vary in their use of structured notes and ICD‐10 codes related to social problems. Approximately 8% of Veterans with primary care services and one in four hospitalized Veterans had engagement with a social worker, which would be necessary to generate structured social work notes.^27^, ^63^ Veterans with low engagement with the healthcare system or low levels of trust prior to hospitalization are more likely to have their social risks unrecognized. To address the need for more universal screening on social risks, policy makers have launched programs to strengthen social screening. Effective January 1, 2023, the Joint Commission created new requirements for healthcare organizations to assess patients' of health related social needs.^64^ In VA, the ACORN tool has been developed as a veteran‐tailored screening and quality improvement initiative and adapted for use at several medical centers.^37^, ^65^ When ACORN is adopted across more VA medical centers, and particularly if administered as part of routine care, it will be able to help address current data by more systematically populating the SOS with population level screening data.

The final SOS score is based only on social domains, an analytic choice conceptually driven by the theory that the relationship of social risks with medical comorbidities is complex and bidirectional. We weighed the benefits of adjustment for all medical risk factors against a more parsimonious adjustment scheme. Our adjusted estimates compare the outcomes of Veterans with social risk factors to those with none as if all groups had similar clinical acuity. The finalSOS, however, was not adjusted for clinical factors to formulate a stand‐alone score. For example, Veterans experiencing food insecurity may have poor diabetes control and complications that increase the risk of hospitalization^66^; to present the association of food insecurity and unplanned care, independent of these clinical factors, is difficult to interpret.

The SOS outperformed the CAN score in positive predictive value and in sensitivity. It is important to note that the CAN tool was designed to predict the outcomes of mortality and all‐cause hospital admission. The primary purpose of this analysis was not to show that social risk factors can achieve marginal improvement over prediction models that are fully saturated with clinical data. Rather, we showed that Veterans who experienced social adversity were at higher risk both medically and socially, and a social risk tool presented new information to inform targeting of supportive services.

### 4.3 Implications for policy, research, and clinical practice

Our study aims to address a need for empirical data on the scope, scale, and impact of Veterans' social factors on their health, and directly connects social risks to outcomes that are patient‐centered and institutional priorities.^67^ The SOS suggests a way to integrate social risk data into clinically useful and actionable information. Critically, social risk data can inform not only the allocation of resources but can help the discharge team incorporate specific interventions and resources into a care plan that centers on a patient's particular needs and challenges.

Prior studies suggest that social care can be effectively deployed to reduce unplanned care. A Chicago hospital program in which social workers managed care transitions to address the non‐medical obstacles to recovery reduced the risk of hospital readmission by 14%.^68^ Social workers assigned to VA PACT teams reduced emergency care and hospital days among high‐CAN‐score Veterans.^46^ Using an integrated SOS could alert primary care staff which Veterans should receive additional social screening and assessment as part of their follow‐up care. Time would be freed up to allow clinicians to focus on providing high‐quality patient care, addressing social needs through VA programs where available, and providing referrals to community organizations and resources if necessary. By considering social risk assessment at admission and planning for discharge with a plan to address social risk factors, the VA can take a proactive approach to preventing unplanned readmissions and treating problems in the primary care before they require emergency care.

Nonetheless, incorporating social risk into everyday care planning could present implementation challenges. Clinicians engaged in the care transition process may benefit from training on the risk that social problems pose to Veterans and the resources available in VA to address social needs. Increased attention to social care may also require investment in social work staff. The VA is adapting a system‐wide understanding of what social determinants and social risk factors are and how they may affect Veteran outcomes. This work highlights how social risk data can be incorporated into transitional care to address Veterans' healthcare outcomes.

## FUNDING INFORMATION

Open access publishing facilitated by The University of Melbourne, as part of the Wiley ‐ The University of Melbourne agreement via the Council of Australian University Librarians.

## Supporting information


**Data S1:** Supporting information.

**Data S2:** Supporting information.
