# Supplementary material for: Using social risks to predict unplanned hospital readmission and emergency care among hospitalized Veterans
Source: Health Serv Res. 2024 Jul 7;60(1):e14353. doi: 10.1111/1475-6773.14353 (PMC11782069; doi:10.1111/1475-6773.14353)
Supplement: Supplementary file 1 — Data S1: Supporting information. [file HESR-60-e14353-s001.docx]

**Using social risks to predict unplanned hospital readmission and emergency care among hospitalized Veterans**

**Online Supplement 1: Model results and sensitivity analyses**

Contents

[Supplement Section S1: Estimates used in main results 2](#_Toc169877756)

[Table S1. Multivariate model estimates used to calculate adjusted predicted risks in main Figure 1 and average marginal effects 2](#_Toc169877757)

[Table S2. Predicted margins and confidence intervals shown in Figure 1 4](#_Toc169877758)

[Table S3. Final Social Risk Score Logistic Model Estimates, Marginal Effects 5](#_Toc169877759)

[Supplement Section 2: Sensitivity Analyses with Mental/Behavioral Domain 6](#_Toc169877760)

[Table S4. Sensitivity analysis: adjusted estimates including mental/behavioral health domain. 6](#_Toc169877761)

[Table S5. Final Social Risk Score Logistic Model Estimates, Marginal Effects: Sensitivity Analysis with Mental/Behavioral Health Domain 9](#_Toc169877762)

[Supplement Section 3: Model testing and validation 11](#_Toc169877763)

[Table S6. Cross-validation of social risk score (SOS) compared to care assessment needs (CAN) at 95^th^ percentile cutoff value 11](#_Toc169877764)

[Table S7. Social risk score (SOS) and Care Assessment Needs (CAN) at 95-percentile frequencies by race and sex 12](#_Toc169877765)

[Figure S3. Sensitivity (true positive percent) is greater for Social-Risk Score (SOS) than Care Assessment Needs (CAN) at 95-percentile cutoff, by race and sex, for predicting [A] hospital readmission and [B] emergency department use. 13](#_Toc169877766)

[Figure S4. Specificity (true negative percent) is similar for Social-Risk Score (SOS) and Care Assessment Needs (CAN) at 95-percentile cutoff, by race and sex, for predicting [A] hospital readmission and [B] emergency department use. 14](#_Toc169877767)

[Table S8. Comparison of Veterans’ characteristics in the top and bottom deciles of Social Risk Score 15](#_Toc169877768)

# Supplement Section S1: Estimates used in main results

## Table S1. Multivariate model estimates used to calculate adjusted predicted risks in main Figure 1 and average marginal effects

|  |  | Unplanned hospital readmission within 30 days of discharge | | | | |  | Emergency department use within 30 days of discharge | | | | |  |
| --- | --- | --- | --- | --- | --- | --- | --- | --- | --- | --- | --- | --- | --- |
|  |  | Estimate (log-odds) | Std. Err. | Pr(>\|z\|) | Average marginal effect (AME) | AME 95% Conf. Int. |  | Estimate (log-odds) | Std. Error | Pr(>\|z\|) | AME | AME 95% Conf. Int. |  |
| Intercept |  | -3.12 | 0.73 | 0.00 |  |  |  | -2.08 | 0.48 | 0.00 |  |  |  |
| Social risk domains | Food insecurity | 0.266 | 0.064 | 0.00 | 0.019 | [0.009, 0.029] |  | 0.218 | 0.043 | 0.00 | 0.040 | [0.024, 0.057] |  |
|  | Legal | 0.248 | 0.075 | 0.00 | 0.018 | [0.006, 0.029] |  | 0.217 | 0.051 | 0.00 | 0.040 | [0.021, 0.060] |  |
|  | Area deprivation | 0.292 | 0.086 | 0.00 | 0.022 | [0.008, 0.035] |  | 0.110 | 0.059 | 0.06 | 0.020 | [-0.002, 0.041] |  |
|  | Financial | 0.204 | 0.047 | 0.00 | 0.014 | [0.007, 0.020] |  | 0.179 | 0.030 | 0.00 | 0.032 | [0.022, 0.043] |  |
|  | Social | 0.131 | 0.041 | 0.00 | 0.009 | [0.003, 0.014] |  | 0.069 | 0.027 | 0.01 | 0.012 | [0.003, 0.022] |  |
|  | Housing | 0.053 | 0.054 | 0.32 | 0.004 | [-0.004, 0.011] |  | 0.161 | 0.034 | 0.00 | 0.029 | [0.017, 0.042] |  |
|  | Violence | 0.101 | 0.080 | 0.21 | 0.007 | [-0.004, 0.018] |  | 0.155 | 0.052 | 0.00 | 0.028 | [0.009, 0.048] |  |
|  | Access to care | -0.136 | 0.072 | 0.06 | -0.009 | [-0.017, 0.000] |  | -0.126 | 0.047 | 0.01 | -0.022 | [-0.037, -0.006] |  |
|  | Nonspecific psychosocial | 0.158 | 0.036 | 0.00 | 0.010 | [0.006, 0.015] |  | 0.086 | 0.022 | 0.00 | 0.015 | [0.007, 0.023] |  |
| Age groups | [55,60) | -0.168 | 0.079 | 0.03 | -0.012 | [-0.022, -0.001] |  | 0.111 | 0.048 | 0.02 | 0.020 | [0.003, 0.037] |  |
|  | [60,65) | -0.151 | 0.066 | 0.02 | -0.011 | [-0.020, -0.002] |  | 0.068 | 0.041 | 0.09 | 0.012 | [-0.002, 0.026] |  |
|  | [65,70) | -0.205 | 0.064 | 0.00 | -0.014 | [-0.023, -0.006] |  | 0.036 | 0.039 | 0.36 | 0.006 | [-0.007, 0.020] |  |
|  | [70,75) | -0.207 | 0.060 | 0.00 | -0.014 | [-0.023, -0.006] |  | 0.052 | 0.037 | 0.16 | 0.009 | [-0.004, 0.022] |  |
|  | [75,80) | -0.183 | 0.063 | 0.00 | -0.013 | [-0.021, -0.004] |  | 0.035 | 0.039 | 0.36 | 0.006 | [-0.007, 0.020] |  |
|  | [80,85) | -0.206 | 0.079 | 0.01 | -0.014 | [-0.025, -0.004] |  | 0.043 | 0.049 | 0.38 | 0.008 | [-0.009, 0.024] |  |
|  | [85,120) | -0.301 | 0.077 | 0.00 | -0.020 | [-0.030, -0.010] |  | -0.022 | 0.047 | 0.65 | -0.004 | [-0.020, 0.012] |  |
| Sex at birth | Male (ref: female) | 0.183 | 0.078 | 0.02 | 0.011 | [0.002, 0.020] |  | 0.009 | 0.045 | 0.83 | 0.002 | [-0.014, 0.017] |  |
| HOSPITAL score | 2 | -0.56 | 0.73 | 0.44 | -0.02 | [-0.09, 0.05] |  | 0.31 | 0.48 | 0.51 | 0.03982 | [-0.07, 0.15] |  |
|  | 3 | 0.03 | 0.73 | 0.97 | 0.00 | [-0.07, 0.07] |  | 0.56 | 0.48 | 0.24 | 0.07754 | [-0.03, 0.18] |  |
|  | 4 | 0.09 | 0.73 | 0.90 | 0.00 | [-0.07, 0.08] |  | 0.66 | 0.48 | 0.16 | 0.09556 | [-0.01, 0.20] |  |
|  | 5 | 0.51 | 0.73 | 0.48 | 0.03 | [-0.04, 0.10] |  | 0.81 | 0.48 | 0.09 | 0.12197 | [0.01, 0.23] |  |
|  | 6 | 0.37 | 0.73 | 0.61 | 0.02 | [-0.05, 0.09] |  | 0.74 | 0.48 | 0.12 | 0.10867 | [0.00, 0.22] |  |
|  | 7 | 0.95 | 0.73 | 0.19 | 0.07 | [0.00, 0.14] |  | 1.05 | 0.48 | 0.03 | 0.16982 | [0.06, 0.28] |  |
|  | 8 | 1.42 | 0.73 | 0.05 | 0.13 | [0.06, 0.20] |  | 1.48 | 0.48 | 0.00 | 0.26547 | [0.16, 0.38] |  |
|  | 9 | 1.09 | 0.73 | 0.14 | 0.09 | [0.01, 0.16] |  | 1.45 | 0.48 | 0.00 | 0.25902 | [0.15, 0.37] |  |
|  | 10 | 1.54 | 0.73 | 0.03 | 0.15 | [0.07, 0.23] |  | 1.50 | 0.48 | 0.00 | 0.27111 | [0.16, 0.38] |  |
|  | 11 | 2.18 | 0.77 | 0.00 | 0.27 | [0.15, 0.40] |  | 1.64 | 0.53 | 0.00 | 0.30495 | [0.15, 0.46] |  |
|  | 12 | 1.6 | 1.3 | 0.23 | 0.16 | [-0.21, 0.54] |  | 1.56 | 1.03 | 0.13 | 0.28559 | [-0.16, 0.73] |  |
|  | 13 | -8.5 | 113.5 | 0.94 | -0.05 | [-0.12, 0.02] |  | -8.55 | 68.98 | 0.90 | -0.13218 | [-0.24, -0.03] |  |
| Notes: AME=average marginal effect, average change in level probability of outcome with one-unit change in exposure.  HOSPITAL score: 13-point scare representing Hemoglobin at discharge, discharge from an Oncology service, Sodium level at discharge, Procedure during the index admission, Index Type of admission (non-elective), number of Admissions during the last 12 months, and Length of stay. | | | | | | | | | | | | | |

## Table S2. Predicted margins and confidence intervals shown in Figure 1

|  | **Unplanned hospital readmission within 30 days** | | | | | | **Any emergency department visit within 30 days** | | | | | |
| --- | --- | --- | --- | --- | --- | --- | --- | --- | --- | --- | --- | --- |
|  | Unadjusted | | | Adjusted (predicted mean) | | | Unadjusted | | | Adjusted (predicted mean) | | |
|  | Mean | CI lower | CI upper | Predicted mean | CI lower | CI upper | Mean | CI lower | CI upper | Predicted mean | CI lower | CI upper |
| Overall sample | 0.074 |  |  |  |  |  | 0.24 |  |  |  |  |  |
| No social risk | 0.052 |  |  | 0.052 |  |  | 0.20 |  |  | 0.21 |  |  |
| Food insecurity | 0.129 | 0.115 | 0.143 | 0.091 | 0.082 | 0.101 | 0.34 | 0.34 | 0.35 | 0.28 | 0.26 | 0.29 |
| Legal | 0.121 | 0.115 | 0.127 | 0.090 | 0.079 | 0.102 | 0.34 | 0.32 | 0.35 | 0.28 | 0.26 | 0.30 |
| Area deprivation | 0.111 | 0.107 | 0.115 | 0.094 | 0.081 | 0.108 | 0.28 | 0.26 | 0.31 | 0.26 | 0.24 | 0.28 |
| Financial | 0.107 | 0.094 | 0.121 | 0.084 | 0.078 | 0.089 | 0.31 | 0.29 | 0.32 | 0.26 | 0.25 | 0.27 |
| Social | 0.107 | 0.100 | 0.114 | 0.080 | 0.075 | 0.085 | 0.29 | 0.28 | 0.31 | 0.25 | 0.24 | 0.26 |
| Housing | 0.107 | 0.102 | 0.113 | 0.076 | 0.070 | 0.083 | 0.32 | 0.30 | 0.33 | 0.26 | 0.25 | 0.27 |
| Violence | 0.103 | 0.090 | 0.116 | 0.080 | 0.069 | 0.091 | 0.31 | 0.30 | 0.31 | 0.27 | 0.25 | 0.29 |
| Access to care | 0.098 | 0.086 | 0.111 | 0.066 | 0.058 | 0.074 | 0.28 | 0.27 | 0.30 | 0.22 | 0.20 | 0.23 |
| Nonspecific psychosocial | 0.091 | 0.085 | 0.096 | 0.078 | 0.075 | 0.082 | 0.27 | 0.24 | 0.29 | 0.25 | 0.24 | 0.25 |

Adjusted estimates are predicted population average from logistic models adjusting for age, sex, and HOSPITAL score (see Table S1).

## Table S3. Final Social Risk Score Logistic Model Estimates, Marginal Effects

|  | 30-day Unplanned Hospital Readmission | | | | | 30-day Emergency Department Visit | | | | |
| --- | --- | --- | --- | --- | --- | --- | --- | --- | --- | --- |
|  | Coeff | Std Err | Average marginal effect (AME) | AME 95%CI |  | Coeff | Std Err | AME | AME 95%CI |  |
| Intercept | -2.910 | 0.027 | 0.028 | [-0.007, 0.015] |  | -1.392 | 0.016 |  |  |  |
| Food insecurity | 0.366 | 0.062 | 0.023 | [0.017, 0.039] |  | 0.270 | 0.043 | 0.051 | [0.035, 0.068] |  |
| Nonspecific psychosocial | 0.341 | 0.035 | 0.021 | [0.018, 0.028] |  | 0.188 | 0.021 | 0.034 | [0.026, 0.042] |  |
| Financial | 0.294 | 0.045 | 0.027 | [0.014, 0.028] |  | 0.221 | 0.029 | 0.041 | [0.030, 0.052] |  |
| Area deprivation | 0.351 | 0.084 | 0.019 | [0.013, 0.042] |  | 0.151 | 0.058 | 0.028 | [0.006, 0.050] |  |
| Legal | 0.258 | 0.073 | 0.017 | [0.008, 0.031] |  | 0.198 | 0.050 | 0.037 | [0.018, 0.056] |  |
| Social support | 0.237 | 0.040 | 0.002 | [0.011, 0.023] |  | 0.126 | 0.026 | 0.023 | [0.014, 0.032] |  |
| Housing | 0.036 | 0.053 | 0.004 | [-0.005, 0.010] |  | 0.152 | 0.034 | 0.028 | [0.016, 0.041] |  |
| Violence | 0.062 | 0.078 | 0.028 | [-0.007, 0.015] |  | 0.118 | 0.051 | 0.022 | [0.003, 0.041] |  |
| Num.Obs. | 51832 |  |  |  |  | 51832 |  |  |  |  |
| AIC | 26791.3 |  |  |  |  | 56415.2 |  |  |  |  |
| BIC | 26871.0 |  |  |  |  | 56494.9 |  |  |  |  |
| Log.Lik. | -13386.636 |  |  |  |  | -28198.601 |  |  |  |  |
| F | 62.028 |  |  |  |  | 78.309 |  |  |  |  |
| RMSE | 0.26 |  |  |  |  | 0.42 |  |  |  |  |

# Supplement Section 2: Sensitivity Analyses with Mental/Behavioral Domain

We did not include mental illness or substance use in our main results but these constructs are often grouped with social risks. Therefore we reported an alternative model in the appendix including this domain. Mental health has a significant association with both outomes, similar in magnitude to the “nonspecific psychosocial” domain (figure S1 and Table S4). Patients with psychiatric comorbidities or self-reported depression are at higher risk for readmission than those without^60,61^ and mental health conditions such as depression and anxiety are associated with early readmissions in prior VA studies.^62^ Younger men with psychotic or affective disorders are disproportionately frequent presenters to the ED.^63^ We use an inclusive definition of mental/behavioral health that results in a high observed prevalence. Further investigation into how members of the care team might use and understand mental-health indicators could inform whether a more specific definition would add value to the social risk taxonomy.

## Table S4. Sensitivity analysis: adjusted estimates including mental/behavioral health domain.

|  |  | Unplanned hospital readmission within 30 days of discharge | | | | |  | Emergency department use within 30 days of discharge | | | | |
| --- | --- | --- | --- | --- | --- | --- | --- | --- | --- | --- | --- | --- |
|  |  | Estimate (log-odds) | Std. Err. | Pr(>\|z\|) | Average marginal effect (AME) | AME 95% Conf. Int. |  | Estimate (log-odds) | Std. Error | Pr(>\|z\|) | AME | AME 95% Conf. Int. |
| Intercept |  | -3.227 | 0.730 | 0.00 |  |  |  | -2.144 | 0.480 | 0.00 |  |  |
| Social risk domains | Food insecurity | 0.265 | 0.064 | 0.00 | 0.019 | [0.009, 0.029] |  | 0.217 | 0.043 | 0.00 | 0.040 | [0.024, 0.057] |
|  | Legal | 0.240 | 0.075 | 0.00 | 0.017 | [0.006, 0.029] |  | 0.212 | 0.051 | 0.00 | 0.039 | [0.020, 0.059] |
|  | Area deprivation | 0.292 | 0.086 | 0.00 | 0.021 | [0.008, 0.035] |  | 0.110 | 0.059 | 0.06 | 0.020 | [-0.002, 0.041] |
|  | Financial | 0.187 | 0.047 | 0.00 | 0.013 | [0.006, 0.019] |  | 0.169 | 0.030 | 0.00 | 0.031 | [0.020, 0.042] |
|  | Social | 0.098 | 0.042 | 0.02 | 0.007 | [0.001, 0.012] |  | 0.050 | 0.027 | 0.06 | 0.009 | [-0.001, 0.018] |
|  | Housing | 0.042 | 0.054 | 0.43 | 0.003 | [-0.004, 0.010] |  | 0.155 | 0.035 | 0.00 | 0.028 | [0.016, 0.041] |
|  | Violence | 0.095 | 0.080 | 0.23 | 0.007 | [-0.005, 0.018] |  | 0.152 | 0.052 | 0.00 | 0.028 | [0.008, 0.047] |
|  | Access to care | -0.148 | 0.072 | 0.04 | -0.009 | [-0.018, -0.001] |  | -0.134 | 0.047 | 0.00 | -0.023 | [-0.038, -0.008] |
|  | Nonspecific psychosocial | 0.156 | 0.036 | 0.00 | 0.010 | [0.006, 0.015] |  | 0.085 | 0.022 | 0.00 | 0.015 | [0.007, 0.023] |
|  | **Mental/Behavioral Health** | **0.169** | **0.037** | **0.00** | **0.011** | **[0.006, 0.016]** |  | **0.095** | **0.023** | **0.00** | **0.017** | **[0.009, 0.025]** |
| Age groups | [55,60) | -0.157 | 0.079 | 0.05 | -0.011 | [-0.021, 0.000] |  | 0.118 | 0.048 | 0.01 | 0.021 | [0.004, 0.038] |
|  | [60,65) | -0.136 | 0.066 | 0.04 | -0.010 | [-0.019, -0.001] |  | 0.077 | 0.041 | 0.06 | 0.014 | [0.000, 0.028] |
|  | [65,70) | -0.186 | 0.064 | 0.00 | -0.013 | [-0.021, -0.004] |  | 0.048 | 0.039 | 0.23 | 0.008 | [-0.005, 0.022] |
|  | [70,75) | -0.188 | 0.060 | 0.00 | -0.013 | [-0.021, -0.005] |  | 0.063 | 0.037 | 0.09 | 0.011 | [-0.002, 0.024] |
|  | [75,80) | -0.161 | 0.063 | 0.01 | -0.011 | [-0.020, -0.003] |  | 0.049 | 0.039 | 0.21 | 0.009 | [-0.005, 0.022] |
|  | [80,85) | -0.170 | 0.080 | 0.03 | -0.012 | [-0.022, -0.001] |  | 0.065 | 0.049 | 0.19 | 0.011 | [-0.006, 0.028] |
|  | [85,120) | -0.254 | 0.078 | 0.00 | -0.017 | [-0.027, -0.007] |  | 0.005 | 0.047 | 0.91 | 0.001 | [-0.015, 0.017] |
| Sex at birth | Male (ref: female) | 0.201 | 0.078 | 0.01 | 0.012 | [0.004, 0.021] |  | 0.019 | 0.045 | 0.67 | 0.003 | [-0.012, 0.019] |
| HOSPITAL score | 2 | -0.54 | 0.73 | 0.46 | -0.021 | [-0.089, 0.048] |  | 0.33 | 0.48 | 0.50 | 0.041 | [-0.065, 0.148] |
|  | 3 | 0.06 | 0.73 | 0.93 | 0.003 | [-0.066, 0.072] |  | 0.58 | 0.48 | 0.23 | 0.080 | [-0.027, 0.186] |
|  | 4 | 0.11 | 0.73 | 0.88 | 0.006 | [-0.063, 0.075] |  | 0.67 | 0.48 | 0.16 | 0.096 | [-0.010, 0.203] |
|  | 5 | 0.54 | 0.73 | 0.46 | 0.033 | [-0.036, 0.102] |  | 0.82 | 0.48 | 0.09 | 0.12 | [0.02, 0.23] |
|  | 6 | 0.39 | 0.73 | 0.59 | 0.022 | [-0.047, 0.092] |  | 0.75 | 0.48 | 0.12 | 0.11 | [0.00, 0.22] |
|  | 7 | 0.97 | 0.73 | 0.18 | 0.073 | [0.003, 0.142] |  | 1.06 | 0.48 | 0.03 | 0.17 | [0.06, 0.28] |
|  | 8 | 1.43 | 0.73 | 0.05 | 0.131 | [0.06, 0.20] |  | 1.48 | 0.48 | 0.00 | 0.27 | [0.16, 0.37] |
|  | 9 | 1.10 | 0.73 | 0.13 | 0.087 | [0.014, 0.160] |  | 1.45 | 0.48 | 0.00 | 0.26 | [0.15, 0.37] |
|  | 10 | 1.55 | 0.73 | 0.03 | 0.150 | [0.07, 0.23] |  | 1.50 | 0.48 | 0.00 | 0.27 | [0.16, 0.38] |
|  | 11 | 2.20 | 0.77 | 0.00 | 0.271 | [0.15, 0.40] |  | 1.65 | 0.53 | 0.00 | 0.31 | [0.15, 0.46] |
|  | 12 | 1.66 | 1.33 | 0.21 | 0.168 | [-0.21, 0.55] |  | 1.59 | 1.03 | 0.12 | 0.29 | [-0.16, 0.74] |
|  | 13 | -8.52 | 113.30 | 0.94 | -0.051 | [-0.120, 0.018] |  | -9 | 69 | 0.90 | -0.13 | [-0.24, -0.02] |
| Notes: AME=average marginal effect, average change in level probability of outcome with one-unit change in exposure.  HOSPITAL score: 13-point scare representing Hemoglobin at discharge, discharge from an Oncology service, Sodium level at discharge, Procedure during the index admission, Index Type of admission (non-elective), number of Admissions during the last 12 months, and Length of stay. | | | | | | | | | | | | |

**Figure S1. Probabilities of unplanned readmission and emergency department use for Veterans with social risks: sensitivity analysis including mental/behavioral health domain.**

**
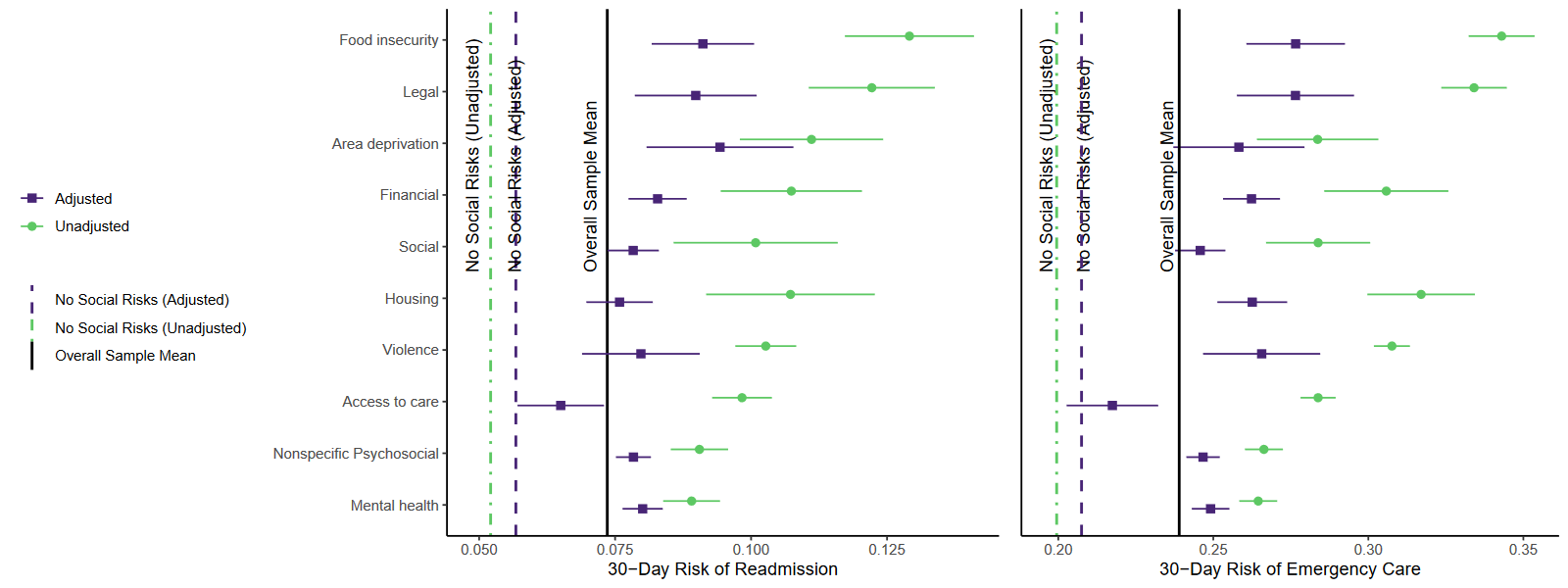
**

## Table S5. Final Social Risk Score Logistic Model Estimates, Marginal Effects: Sensitivity Analysis with Mental/Behavioral Health Domain

|  | 30-day Unplanned Hospital Readmission | | | | | 30-day Emergency Department Visit | | | | |
| --- | --- | --- | --- | --- | --- | --- | --- | --- | --- | --- |
|  | Coeff | Std Err | Average marginal effect (AME) | AME 95%CI |  | Coeff | Std Err | AME | AME 95%CI |  |
| Intercept | -2.97 | 0.03 |  |  |  | -1.424 | 0.017 |  |  |  |
| Food insecurity | 0.361 | 0.062 | 0.028 | [0.017, 0.038] |  | 0.268 | 0.043 | 0.05095 | [0.034, 0.068] |  |
| Nonspecific psychosocial | 0.342 | 0.035 | 0.023 | [0.018, 0.028] |  | 0.188 | 0.021 | 0.03395 | [0.026, 0.042] |  |
| Financial | 0.266 | 0.046 | 0.019 | [0.012, 0.026] |  | 0.206 | 0.029 | 0.03823 | [0.027, 0.049] |  |
| Area deprivation | 0.349 | 0.084 | 0.027 | [0.012, 0.041] |  | 0.150 | 0.058 | 0.02785 | [0.006, 0.050] |  |
| Legal | 0.238 | 0.073 | 0.018 | [0.006, 0.029] |  | 0.187 | 0.050 | 0.03506 | [0.016, 0.054] |  |
| Social support | 0.198 | 0.041 | 0.014 | [0.008, 0.020] |  | 0.105 | 0.026 | 0.01919 | [0.010, 0.029] |  |
| Housing | 0.020 | 0.053 | 0.001 | [-0.006, 0.008] |  | 0.143 | 0.034 | 0.02646 | [0.014, 0.039] |  |
| Violence | 0.049 | 0.078 | 0.003 | [-0.007, 0.014] |  | 0.110 | 0.051 | 0.02024 | [0.001, 0.039] |  |
| Mental/Behavioral Health | 0.199 | 0.036 | 0.014 | [0.009, 0.018] |  | 0.105 | 0.022 | 0.01889 | [0.011, 0.027] |  |
| Num.Obs. | 51832 |  |  |  |  | 51832 |  |  |  |  |
| AIC | 26755.5 |  |  |  |  | 56390.1 |  |  |  |  |
| BIC | 26844.1 |  |  |  |  | 56478.7 |  |  |  |  |
| Log.Lik. | -13367.749 |  |  |  |  | -28185.074 |  |  |  |  |
| F | 59.507 |  |  |  |  | 72.579 |  |  |  |  |
| RMSE | 0.26 |  |  |  |  | 0.42 |  |  |  |  |

Notes: Coefficients represent log-odds increase in probability associated with having the social risk, conditional on other social risks. Average marginal effects represent the level increase in probability associated with the social risk, conditional on other social risks. To calculate the final social risk score, log-odds coefficients were summed for each social risk domain, the score was imputed for each Veteran, and a percentile-rank score calculated.

Figure S2. Risks of readmission and emergency care plotted against social risk score (SOS) and care assessment needs (CAN) percentiles: alternative models including mental/behavioral health.


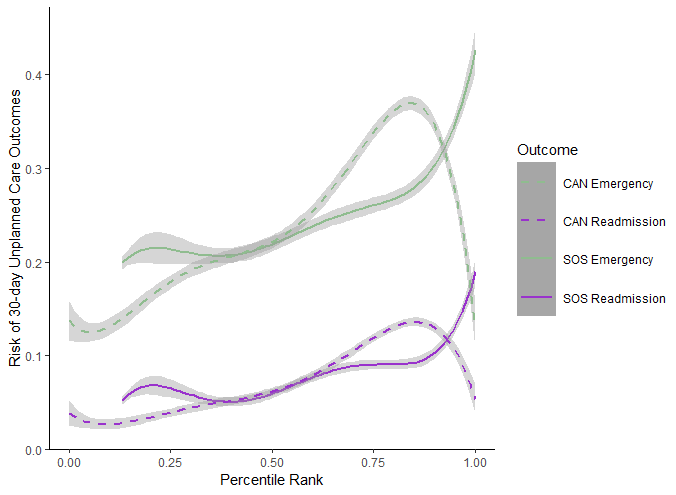


# Supplement Section 3: Model testing and validation

## Table S6. Cross-validation of social risk score (SOS) compared to care assessment needs (CAN) at 95^th^ percentile cutoff value

Table S6, below, shows a cross-validation of the social risk score (SOS) model 5-group K-fold validation. The sample was randomly partitioned into 5 sections. For each iteration, the readmission and emergency department models were estimated on the K-1 sections and weights re-calculated. We then used those weights to impute the SOS percentile score for the observations in the “left-out” section. To assess model performance, we calculated the sensitivity of the ≥95 threshold as a positive signal, defined as the percent of patients with positive signal (score ≥95), conditional on having the unplanned-care outcome. The procedure was repeated over 5 iterations. We found that the model performed well on the left-out groups.

|  | Full data (in-model) | K-fold | | | | |
| --- | --- | --- | --- | --- | --- | --- |
| Model diagnostic |  | 1 | 2 | 3 | 4 | 5 |
| **Hospital readmission 30 days (overall percent)** | 7.4% | 7.3% | 7.0% | 7.4% | 7.5% | 7.6% |
| SOS≥95 readmission sensitivity | 10.6% | 9.9% | 9.7% | 10.1% | 11.3% | 12.2% |
| CAN≥95 readmission sensitivity | 3.0% | 2.6% | 3.4% | 2.3% | 3.3% | 3.2% |
|  |  |  |  |  |  |  |
| **ED use 30 days (overall percent)** | 23.9% | 24.1% | 23.4% | 24.3% | 23.4% | 24.3% |
| SOS≥95 ED sensitivity | 8.1% | 8.3% | 7.6% | 7.9% | 8.3% | 8.6% |
| CAN≥95 ED sensitivity | 2.3% | 2.4% | 2.6% | 1.9% | 2.7% | 2.2% |

## Table S7. Social risk score (SOS) and Care Assessment Needs (CAN) at 95-percentile frequencies by race and sex

|  | American Indian or Alaska Native | Asian | Black | Native Hawaiian or Other Pacific Islander | White | More than one race | Unknown race | Female | Male | Total |
| --- | --- | --- | --- | --- | --- | --- | --- | --- | --- | --- |
| Observations, count | 545 | 195 | 9377 | 300 | 38332 | 548 | 2535 | 3228 | 48604 | 51832 |
| SOS≥95, count | 93 | <10 | 722 | 22 | 1601 | 51 | 142 | 166 | 2473 | 2639 |
| CAN≥95, count | 18 | <10 | 361 | <10 | 1098 | 24 | 142 | 90 | 1570 | 1660 |
| 30-day hospital readmission, percent | 10.8% | 3.1% | 7.0% | 7.0% | 7.5% | 8.0% | 6.6% | 6.1% | 7.4% | 7.4% |
| 30-day emergency department use, percent | 27.3% | 16.4% | 26.2% | 24.7% | 23.4% | 27.4% | 21.7% | 23.1% | 24.0% | 23.9% |

## Figure S3. Sensitivity (true positive percent) is greater for Social-Risk Score (SOS) than Care Assessment Needs (CAN) at 95-percentile cutoff, by race and sex, for predicting [A] hospital readmission and [B] emergency department use.

Figure S1 Notes: CAN=Care Assessment Needs Score, SOS=Social Risk Score, ED=emergency department. Sensitivity defined as the percent of patients who had score ≥95 at the time of discharge, among those who then experienced the outcome within 30 days from discharge.

## Figure S4. Specificity (true negative percent) is similar for Social-Risk Score (SOS) and Care Assessment Needs (CAN) at 95-percentile cutoff, by race and sex, for predicting [A] hospital readmission and [B] emergency department use.

Figure S1 Notes: CAN=Care Assessment Needs Score, SOS=Social Risk Score, ED=emergency department. Specificity is the percent of patients whose score on the scale was <95, among those that did not experience the outcome within 30 days. Data are excluded for groups with cell counts <=10.

## Table S8. Comparison of Veterans’ characteristics in the top and bottom deciles of Social Risk Score

|  | 10th Percentile | 90th Percentile | SMD |
| --- | --- | --- | --- |
| No. of observations | 19,131 | 5,529 |  |
| Age (mean (SD)) | 69.13 (13.19) | 63.10 (14.92) | 0.428 |
| Sex |  |  |  |
| Male (%) | 18,107 (94.6) | 5,160 (93.3) | 0.056 |
| Female (%) | 1,024 (5.4) | 369 (6.7) |  |
| Race (%) |  |  | 0.35 |
| American Indian or Alaska Native | 152 (0.8) | 142 (2.6) |  |
| Asian | 104 (0.5) | 16 (0.3) |  |
| Black or African American | 2,397 (12.5) | 1,317 (23.8) |  |
| Native Hawaiian or Other Pacific Islander | 103 (0.5) | 35 (0.6) |  |
| White | 15,294 (79.9) | 3,660 (66.2) |  |
| More than one race | 197 (1.0) | 88 (1.6) |  |
| Unknown | 884 (4.6) | 271 (4.9) |  |
| VA priority (%) |  |  | 0.323 |
| Low-Income (5,7) | 4,695 (24.5) | 1,781 (32.2) |  |
| Missing | 9 (0.0) | 2 (0.0) |  |
| Other (6,8) | 2,401 (12.6) | 270 (4.9) |  |
| Other Disability (4) | 377 (2.0) | 217 (3.9) |  |
| Service-Connected Disability (1,2) | 9,559 (50.0) | 2,677 (48.4) |  |
| Wartime Service (3) | 2,090 (10.9) | 582 (10.5) |  |
| RUCA_groups3 (%) |  |  | 0.273 |
| Commuters | 2,896 (15.1) | 562 (10.2) |  |
| Isolated rural | 1,520 (7.9) | 252 (4.6) |  |
| Large rural city | 3,649 (19.1) | 929 (16.8) |  |
| Small rural town | 1,812 (9.5) | 421 (7.6) |  |
| Urban | 9,254 (48.4) | 3,365 (60.9) |  |
| HOSPITAL score (mean (SD)) | 3.84 (1.74) | 5.37 (1.94) | 0.828 |

Table S8 shows characteristics of Veterans with highest and lowest social risk. Note that the bottom decile includes more than 10% of Veterans with no social risk factors. The comparison elucidates who the highest risk Veterans are to spur thinking about targeting and prioritization of supportive services.
